# Determining Sex-Based Differences in Inflammatory Response in an Experimental Traumatic Brain Injury Model

**DOI:** 10.3389/fimmu.2022.753570

**Published:** 2022-02-09

**Authors:** Michael C. Scott, Karthik S. Prabhakara, Andrew J. Walters, Scott D. Olson, Charles S. Cox

**Affiliations:** Department of Pediatric Surgery, University of Texas Health Science Center at Houston, Houston, TX, United States

**Keywords:** traumatic brain injury, sex-based differences, neuroinflammation, blood-brain barrier, neurologic injury

## Abstract

**Introduction:**

Traumatic brain injury is a leading cause of injury-related death and morbidity. Multiple clinical and pre-clinical studies have reported various results regarding sex-based differences in TBI. Our accepted rodent model of traumatic brain injury was used to identify sex-based differences in the pathological features of TBI.

**Methods:**

Male and female Sprague-Dawley rats were subjected to either controlled-cortical impact (CCI) or sham injury; brain tissue was harvested at different time intervals depending on the specific study. Blood-brain barrier (BBB) analysis was performed using infrared imaging to measure fluorescence dye extravasation. Microglia and splenocytes were characterized with traditional flow cytometry; microglia markers such as CD45, P2Y12, CD32, and CD163 were analyzed with t-distributed stochastic neighbor embedding (t-SNE). Flow cytometry was used to study tissue cytokine levels, and supplemented with ELISAs of TNF-⍺, IL-17, and IL-1β of the ipsilateral hemisphere tissue.

**Results:**

CCI groups of both sexes recorded a higher BBB permeability at 72 hours post-injury than their respective sham groups. There was significant difference in the integrated density value of BBB permeability between the male CCI group and the female CCI group (female CCI mean = 3.08 x 108 ± 2.83 x 107, male CCI mean = 2.20 x 108 ± 4.05 x 106, p = 0.0210), but otherwise no differences were observed. Traditional flow cytometry did not distinguish any sex-based difference in regards to splenocyte cell population after CCI. t-SNE did not reveal any significant difference between the male and female injury groups in the activation of microglia. Cytokine analysis after injury by flow cytometry and ELISA was limited in differences at the time point of 6 hours post-injury.

**Conclusion:**

In our rodent model of traumatic brain injury, sex-based differences in pathology and neuroinflammation at specified time points are limited, and only noted in one specific analysis of BBB permeability.

## Introduction

Traumatic brain injury (TBI) is one of the leading causes of death and disability related to trauma in the United States. In 2014, the CDC estimated that TBI accounted for 2.87 million Emergency Department visits, 288,000 hospitalizations, and 56,800 deaths (1). The Global Burden of Disease Study found that the age-adjusted incidence rate of TBI in 2016 was 369 per 100,000. The global prevalence of individuals living with TBI-related disability was 759 per 100,000; TBI alone accounted for 8.1 million years lived with disability in 2016 (2). TBI is characterized by two phases of injury: primary injury and secondary injury. Primary injury is the initial insult *via* physical impact and damage to brain tissue. Secondary injury from TBI is characterized by an increase in neuronal excitotoxicity and progression of neuroinflammation (3).

Current treatment modalities for severe TBI focus on control of intracranial hypertension, maintenance of adequate cerebral perfusion pressure, and supportive care. Treatment options include surgical decompression, hyperosmolar therapy, temporizing ventilator strategies, optimization of nutrition, infection prophylaxis, and anti-epileptic medication for seizure prophylaxis (4). Despite the benefits of these options, few truly address slowing or halting the progression of TBI. Thus, research in TBI has emphasized understanding its pathophysiology in order to develop targeted therapies that limit injury progression and promote resolution (3).

Sexual dimorphism may influence the progression of TBI and may need to be considered in pre-clinical and clinical studies. Some clinical studies have suggested that there is a difference in outcomes with the female sex deriving some benefit in mortality and complication risk (5); other clinical studies have indicated that there is no significant difference (6). TBI severity may influence these observed differences (7). Pediatric studies have suggested that females may have some survival advantage compared to males after sustaining a TBI. Improved survival in pre- and post-pubescent stage females has been observed in retrospective studies of severe TBI (8, 9). Some retrospective studies allude to a variance in post-injury functional and psychological outcomes. For example, males and females who have sustained a TBI report different clinical symptoms, such as sleep disturbances and noise sensitivity (10, 11). Scott et al. found that psychological issues after TBI may differ in males and females as well; males were more likely to report substance abuse, and females were more likely to report experiencing anxiety or depression (11). In contrast to studies reporting differences, a recently published retrospective study of data from the Chinese Head Trauma Data Bank indicated no significant difference between male and female adult patients in mortality or long-term unfavorable outcomes after acute TBI (12).

Pre-clinical studies have demonstrated potential variability in the secondary injury of TBI. Doran et al. found that the increase in both peripheral myeloid influx and resident microglia proliferation in the injured cerebral hemisphere after controlled-cortical impact (CCI) injury was higher in male rats compared to female rats. Bromberg et al. found that Iba-1 staining of microglia, was higher in male rats in the paraventricular nucleus 7 days after sustaining fluid-percussion injury (FPI), indicating a higher number of microglia (13, 14). Armstead et al. have indicated that cerebral autoregulation may have sex-based differences (15–17). Different mechanisms to induce TBI are used in animal studies, which can influence the results. In a review of animal studies, 55% of studies using CCI as an injury mechanism reported females having better outcomes in post-injury evaluation such as behavioral and motor function studies. When using a closed-head injury (CHI) mechanism, 31% of studies showed females with improved outcomes in post-injury evaluations like cytoskeletal degradation, reactive astrogliosis, and cortical blood flow. Studies using fluid-percussion injury (FPI) reported females having worse survival and regulation of cell apoptosis in 60% of studies. However, these apparent differences may be in part depend on the outcomes measured (7).

**Figure 1:**
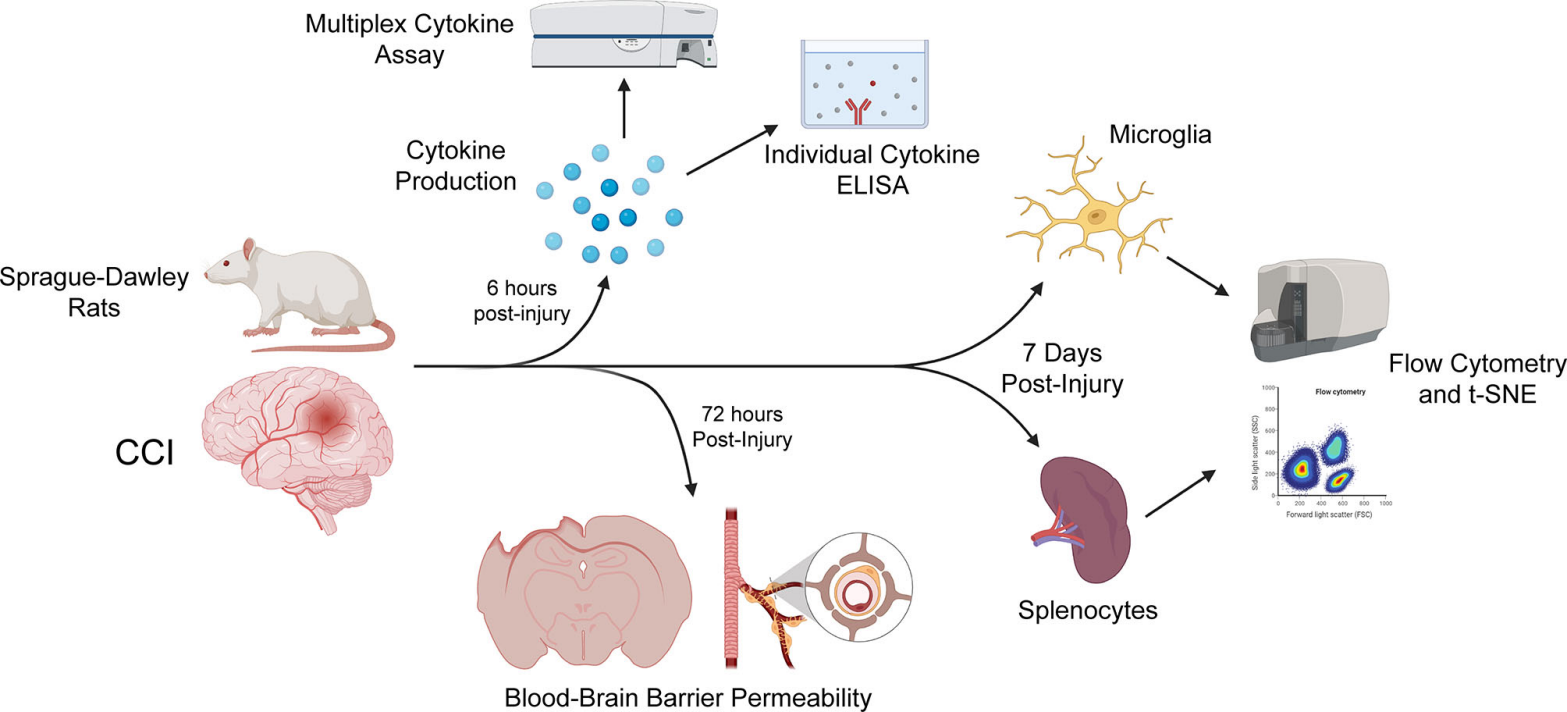
Time points post-injury for each study. All rats underwent either sham or CCI injury. For cytokine studies of brain tissue, including individual ELISAs and a multiplex cytokine assay (performed using the LSR-II flow cytometer [BD Bioscience]), rats were sacrificed 6 hours after injury, to capture the peak of cytokine production. Rats were sacrificed 72 hours post-injury for our assessment of blood-brain barrier permeability. Flow cytometry and t-SNE of microglia and splenocyte immunophenotype using the Beckman-Coulter Gallios flow cytometer was performed 7 days after injury. Created with BioRender.com.

After review of the information above and other studies (**Tables 1**, **2**), we used our CCI model with our specific methodological outcome measures to uncover any sex-based differences in neuroinflammation. Experimental groups of male and female Sprague-Dawley rats sustaining either CCI or sham injury were compared. The progression of neuroinflammation at various time intervals after injury was evaluated based on the following features: microglial and splenocyte cell analysis with flow cytometry, blood-brain barrier (BBB) permeability, and tissue cytokine analysis (**Figure 1**).

**Table 1:** Human studies of sex-based differences in traumatic brain injury.

Study title	Author and year	Study type	Results and findings
The effect of gender on patients with moderate to severe head injuries.	Berry et al., (5).	Retrospective study Age separated	Females had lower risk of mortality and complications. Premenopausal and postmenopausal women had a lower risk of mortality than age matched men. No difference in mortality between premenopausal women and age matched men
Does sexual dimorphism influence outcome of traumatic brain injury patients? The answer is no!	Coimbra et al., (6)	Retrospective study Case controlled	No significant difference in overall outcome or subset analysis. Excluding patients older than 50 years did not change result.
Sex differences in traumatic brain injury: what we know and what we should know.	Gupte et al., (7)	Analysis of literature review	Overall, human studies report worse outcomes in women than men, animal studies report better outcomes in females than males. A greater percentage of human studies show women have better outcomes in moderate-severe TBI.
Gender impacts mortality after traumatic brain injury in teenagers.	Ley et al., (8)	Retrospective study Pediatric, age separated	Lower mortality rate in 0–12-year-old patients than 12-18-year-olds. No difference between males and females in 0–12-year-olds. Reduced mortality in females in 12-18-year-olds.
Use of a pediatric cohort to examine gender and sex hormone influences on outcome after trauma	Phelan et al., (9)	Retrospective study Pediatric, age separated	0-8-year old and 8.1-14.5-year-old patients had equivalent survival rates between genders across all severities. 14.6-20-year-olds had a significantly improved survival rate for women across all subgroups, more pronounced with increasing injury severity score.
A comparison of adult outcomes for males compared to females following pediatric traumatic brain injury	Scott et al., (10)	Observational study of adults with childhood history of TBI	Patients with childhood TBI had high rates of problem behaviors compared to controls. Females more likely to report a history of internalizing problems. Males more likely to report externalizing problems.
Gender differences in self-reported long-term outcomes following moderate to severe traumatic brain injury	Colantonio et al., (11)	Retrospective study	Difference between self-reported symptoms men and women in long term outcomes and symptoms related to daily functioning.
Chinese Head Trauma Data Bank: Effect of gender on the outcome of patients with acute traumatic brain injury	Chinese Head Trauma Study Collaborators (12)	Observational study	No significant difference in the outcome of patients with acute TBI between men and women
Acute serum hormone levels: characterization and prognosis after severe traumatic brain injury	Wagner et al., (18)	Observational study	Acute serum hormone levels were significantly altered after severe TBI. Increased hormone levels were associated with increased mortality and worse global outcomes.
Pituitary function within the first year after traumatic brain injury or subarachnoid hemorrhage	Tölli et al., (19)	Observational study	Perturbations in pituitary function were frequent early after the event but declined after the first year. No relationship seen between hormonal levels and injury variables.
Chronic hypopituitarism after traumatic brain injury: risk assessment and relationship to outcome	Bavisetty et al., (20)	Observational study	TBI patients with hormonal deficiencies had worse disability rating scale, greater rates of depression, worse quality of life, emotional well-being, and general health.

**Table 2:** Pre-clinical animal studies of sex-based differences.

Study	Author and year	Study details	Results and Findings
Sex-dependent pathology in the HPA axis at a sub-acute period after experimental traumatic brain injury	Bromberg et al., (13)	Rat, mild fluid percussion injury	Males had injury induced neuroinflammation and astrocytosis compared with sex match shams, females did not. Glucocorticoid receptor protein levels elevated in females compared with sex match shams, males did not.
Both estrogen and progesterone attenuate edema formation following diffuse traumatic brain injury in rats.	O’Connor et al., (21)	Rat, weight drop impact	Male rats had an increase in BBB permeability compared to female rats after neurologic injury. Administration of estrogen/progesterone reduced BBB permeability.
Sex Differences in Thermal, Stress, and Inflammatory Responses to Minocycline Administration in Rats with Traumatic Brain Injury.	Taylor et al., (22)	Rat, CCI	At 35 days post-injury, ovariectomized female rats had a greater expression of IL-1β and IL-6 in the ipsilateral hippocampus, but males had a greater expression of TNF-α.
Gender influences outcome of brain injury: progesterone plays a protective role	Roof et al., (23)	Rat, bilateral cortical contusions Male vs. pseudo-pregnant vs. normally cycling females vs ovariectomized ± estrogen and/or progesterone replacement	Males and normally cycling females had a significant increase in edema. Pseudopregnant females had no significant increase in edema. All three groups were significantly different from one another. Replacement of estrogen in ovariectomized rats did not alter response, while replacement of progesterone did.
Estrogen-related gender difference in survival rate and cortical blood flow after impact-acceleration head injury in rats	Roof et al., (24)	Rat, Marmarou impact-acceleration head injury. Ovariectomized females and males ± estradiol replacement	Significantly more females survived injury. Females showed less reduction and better recovery of cortical blood flow. Postinjury cortical blood flow was higher in female and male rats with estradiol injections.
Neuropathological protection after traumatic brain injury in intact female rats versus males or ovariectomized females	Bramlett et al., (25)	Rat, fluid percussion injury. Intact females, ovariectomized females, and males compared.	Intact females had smaller cortical contusion compared to males. Non-proestrous group was significantly different from ovariectomized females. Overiectomzed females had larger areas of damage compared to intact females, similar to males.
Evaluation of estrous cycle stage and gender on behavioral outcome after experimental traumatic brain injury	Wagner et al., (26)	Rat, CCI Females proestrous or non-proestrous.	No significant difference between females regardless of estrous cycle. Females performed significantly better than males on motor function tasks.
Sex differences in acute neuroinflammation after experimental traumatic brain injury are mediated by infiltrating myeloid cells	Doran et al., (14)	Mice, CCI	Males had an influx of peripheral myeloid cells, followed by proliferation of microglia. Females had improved motor function at 1 day.
Male and female mice exhibit divergent responses of the cortical vasculature to traumatic brain injury	Jullienne et al., (27)	Mice, CCI	No difference between males and females in lesion volume, neurodegeneration, blood brain barrier alteration, and microglial activation. Females had more astrocytic hypertrophy and heme-oxygenase-1 induction at one day post injury. Males exhibited increased endothelial activation and expression of β-catenin. 7 days: males had an increase in number of vessels and vessel complexity.
Sex-Dependent Macromolecule and Nanoparticle Delivery in Experimental Brain Injury.	Bharadwaj et al., (28)	Mice, CCI	Female mice at random stages of estrous cycle had an increase in macromolecular tracer accumulation, indicating an increase in BBB permeability compared to males at 3 hours and 24 hours. There was no sex-based difference in neuroglial response.
Blood-brain barrier breakdown and edema formation following frontal cortical contusion: does hormonal status play a role?	Duvdevani et al. (29)	Mice, CCI	Using Evans blue dye, no sex-based difference in BBB permeability was observed. Hormonal status did not have an effect on BBB permeability in male or female rats.
Cytoskeletal protein degradation and neurodegeneration evolves differently in males and females following experimental head injury	Kupina et al., 2003 (30)	Mice, weight drop impact	Male peak protein degradation and neurodegeneration at 3 days, females at 14 days after injury.
Lack of a gender difference in post-traumatic neurodegeneration in the mouse controlled cortical impact injury model	Hall et al., (31)	Mice, CCI	A focal CCI showed no gender difference.
Sexual dimorphism in the inflammatory response to traumatic brain injury.	Villapol et al., (32)	Mice, CCI	Male mice had a more rapid influx and activation of microglia to CCI lesion site compared to females. Differences were indistinguishable at 1-week post injury.
Impaired cerebral blood flow autoregulation during posttraumatic arterial hypotension after fluid percussion brain injury is prevented by phenylephrine in female but exacerbated in male piglets by extracellular signal-related kinase mitogen-activated protein kinase upregulation	Armstead et al., (15)	Pig, fluid percussion brain injury Intervention: phenylephrine	Pial artery dilation was impaired more in males than females. Phenylephrine decreased impairment of pial artery dilation in females but caused vasoconstriction in males. Cerebral blood flow, cerebral perfusion pressure, and autoregulatory index decreased in males, less in females. Phenylephrine reduced ERK MAPK upregulation in females, increased upregulation in males.
TBI sex dependently upregulates ET-1 to impair autoregulation, which is aggravated by phenylephrine in males but is abrogated in females	Armstead et al., (16)	Pig, fluid percussion injury Intervention: phenylephrine and antagonists/scavengers to elucidate mechanism.	Endothelin-1, activated oxygen, and ERK MAPK released in males than females, contributing to impaired autoregulation during hypotension after TBI.
Adrenomedullin reduces gender-dependent loss of hypotensive cerebrovasodilation after newborn brain injury through activation of ATP-dependent K channels	Armstead et al., (17)	Pig, fluid percussion injury Intervention: adrenomedullin	Impaired potassium channel. Adrenomedullin induced pial artery dilation was y greater in female than male piglets. Hypotensive pial artery dilation was blunted to a greater degree in males than females. Topical pretreatment with adrenomedullin reduced the loss of hypotensive pial artery dilation in both genders, but protection was significantly greater in males.

## Materials and Methods

### Animals

All animal experiments were approved by the institutional Animal Welfare Committee of the University of Texas Health Science Center at Houston, TX, USA, in compliance with the NIH Guide for the Care and Use of Laboratory Animals. All experiments complied with the standards of the American Association for the Accreditation of Laboratory Animal Care. The approved protocol number was AWC‐18‐0121. Sprague Dawley rats (Envigo Labs, Indianapolis, Indiana) were used in this series of experiments. A total of 62 rats (5-7 weeks) were used in the study; 31 were males (225-250g) and 31 were females (200-225g). Rats were housed in same-sex pairs in rat microisolators under 12-hour light/dark cycles. Housing conditions were temperature controlled; water and standard rodent laboratory chow were available ad libitum. Rats were randomized to CCI versus sham injury prior to use. Rats were weighed immediately prior to injury. The rats were controlled for age. There was a significant difference in weight; female rats were smaller in weight on average compared to male rats. This will be discussed further in the results. We controlled rats for age to ensure they were at a similar stage of brain development; controlling rats by weight would likely require the use of older female rats.

### Controlled Cortical Impact Injury Model

We used a previously established protocol of controlled cortical impact injury (CCI) to model TBI in rats (33–36). Briefly, animals were anesthetized with 4% isoflurane and oxygen at a flow rate 5 Liters/minute in a vented chamber and then maintained at 1% to 2% isoflurane and oxygen 3 Liters/minute for the duration of the procedure. Next, the animal was secured on a stereotactic frame. The surgical site on the scalp was prepared with alcohol and iodine solution. Subcutaneous 0.25% bupivacaine was administered for local anesthesia. A midline scalp incision was then made; the right‐sided musculature and soft tissue were bluntly dissected away for exposure of the calvarium. Using a dental drill, a 7‐mm diameter craniectomy was performed between the right coronal and lambdoid sutures. A CCI device (Impact One Stereotaxic Impactor, Leica Microsystems, Buffalo Grove, Illinois) was utilized to administer a standardized and unilateral severe brain injury. Injury parameters included a depth of 2.7 mm, impact velocity of 5.6 m/s, and a dwell time of 150 microseconds using a 4‐mm diameter impactor tip to the parietal association cortex. Immediately after the injury, the incision was closed using sterile wound clips and animals were allowed to recover in newly cleaned microisolator cages provided by the University Center for Laboratory Medicine and Care (CLAMC). Sham injuries were performed by anesthetizing the animals, making the midline incision, and separating the skin, connective tissue and aponeurosis from the cranium. The incision was closed using sterile wound clips. In our lab, this has been our traditional method of creating a sham group. We are of the view that the addition of a sham craniectomy may induce underlying neuroinflammation, which is supported by recent studies (37, 38). Also, the primary intention of this study is to focus on differences between sexes in a true TBI, and not necessarily the sham injury.

### Blood Brain Barrier Permeability Measurement

Blood brain barrier (BBB) permeability was measured using a far-red dye (Alexa Fluor 680, ThermoFisher Scientific, MA, USA) bioconjugated to a 10 kDa dextran molecule, as previously reported by our group and used in several additional studies (39–42). CCI and sham injuries were induced as previously described. In this set of injuries, rats were sacrificed 72 hours post-injury. This time point was chosen to keep this study consistent with our prior studies of BBB permeability after injury (41, 43–45). At 72 hours after injury and 30 minutes prior to euthanasia, 0.5 mL of 1 mg/mL Alexa Fluor 680 dye was intravenously injected in rats *via* tail vein. Following exsanguination and 4% paraformaldehyde cardiac perfusion, the brains were harvested, sliced in 2 mm coronal sections and scanned for Alexa Fluor 680 signal using an Odyssey laser scanner (LI-COR Biosciences, NE, USA) in the 700 nm emission channel; auto-fluorescence was visualized in the nonspecific 800 nm emission channel. Quantitative measurements of dye extravasation were determined for each brain by using a uniform region of interest and measuring mean intensity and the mean integrated density of the slices using a threshold to exclude background and low intensity autofluorescence (ImageJ).

### Microglia Flow Cytometry Immunophenotyping

Upon sacrifice, 7 days after CCI injury, brains were harvested and processed as described previously (41, 46, 47). The 7-day time point was chosen to evaluate microglia to allow for comparison to our previous studies (41). This time point has been viewed as a point of peak microglial activation after CNS injury (48). Briefly, the ipsilateral and contralateral side of the brain was processed separately using the Adult Neural Tissue Dissociation Kit GentleMACS Dissociator (Miltenyi Biotech), using manufacturer protocol. Following enzyme digestion, the myelin was removed by Percoll centrifugation. Approximately 1 x 10^6^ cells from the cerebral tissue digestion were used for flow cytometry. A multicolor flow cytometry microglia/myeloid cell panel recently developed in our lab was used to stain and identify microglia and their immunophenotype (46). Our staining method identified the following microglial markers: P2Y12, CD11bc, CD45, CD32, CD163, RT1b. Aliquots of the cerebral tissue digestion were placed BD Trucount tubes (BD Biosciences) to determine absolute cell counts. Traditional flow cytometry analysis was also performed with FlowJo vr10.6.1 (FlowJo, LLC, Ashland, Oregon). In order to identify P2Y12 positive microglia, live cells were initially gated by P2Y12 expression and then gated on CD11bc and CD45. CD11‐positive cells were then gated on phenotypic markers CD32, RT1B, and CD163. To ensure identification of all microglia and myeloid cells, live cells were gated on CD11bc and CD45 without P2Y12. CD11bc‐positive cells were then gated on phenotypic markers CD32, RT1B, and CD163. Data for microglial cells was acquired by a Gallios Flow Cytometer (Beckman Coulter).

### Splenocyte Flow Cytometry Immunophenotyping

Spleens were harvested from animals at the time of euthanasia, 7 days following injury. Splenocytes were isolated as previously described (49). Briefly, the spleen was washed in 10 mL of PBS, homogenized using a gentleMACS Dissociator (Miltenyi Biotech). The cells were then filtered through a 70‐μm filter and centrifuged at 400g for 5 minutes. Next, flow cytometry was performed to characterize lymphoid and myeloid cell populations. The antibody panel consisted of the following cell surface markers: anti‐CD3‐FITC, anti‐CD25‐PE, anti‐CD8a‐PerCP, anti‐CD11bc‐PECy7, anti‐RT1B‐APC, anti‐CD4‐APCCy7, anti‐CD45RA‐V450. All antibodies were purchased from Beckman Coulter. Myeloid cells were identified as CD11bc‐positive, CD3‐negative, CD45RA‐negative cells. B cells were identified as CD45RA‐positive, CD3‐negative, CD11bc‐negative cells. T cells were identified as CD3‐positive, CD11bc‐negative, CD45RA‐negative cells. Further T‐cell subsets were identified using the CD4, CD8, and CD25 markers. Data for the splenocyte samples was acquired by a Gallios Flow Cytometer (Beckman Coulter) using Kaluza acquisition software. Subsequent data analyses were completed utilizing FlowJo (vr10.6.1).

### T‐Distributed Stochastic Neighbor Embedding Analysis

t‐Distributed stochastic neighbor embedding (t‐SNE) is a tool that analyzes flow cytometry data, by projecting all data points onto a multi-dimensional graph plot. t-SNE forms a “heat-map” of all cells that were analyzed by flow cytometry. We perform t-SNE to better characterize specific populations of cells and other features such as their morphometry and expression of activation markers. The t-SNE analysis was performed with FlowJo (vr10.6.1). Briefly, live cells were gated on all samples. Within each sample, live cell events were randomly downsampled to 3000 events; analysis was run on an equal number of events per sample. The individual sample files were concatenated to link them together into a single‐standard file. t‐SNE was run using the FlowJo plugin, which included all fluorophores previously listed. Unique t‐SNE was created for both splenocyte and microglia panels with all samples and groups derived from the same t-SNE run, for consistency.

### Inflammatory Cytokine Cytometric Profile

Finally, we studied the expression of inflammatory cytokines in brain tissue. The brains were harvested at 6 hours post injury from rats following euthanasia. This time point typically represents peak pro-inflammatory cytokine expression post-injury (48, 50). For each brain, the ipsilateral hemisphere was homogenized in RIPA buffer (Alfa Aesar) containing protein inhibitor cocktail (Thermo Scientific) using PT-3100 Polytron homogenizer (Kinamatia AG). The protein was quantified by BCA kit (Thermo Scientific). Tissue lysate at a concentration of 1mg/mL was run on LEGENDplex™ Rat Inflammation Panel (13-plex) kit, using manufacturer protocol and an LSR-II flow cytometer (BD Biosciences). For further verification of our multiple cytokine panel, more accurate and sensitive ELISAs (Biolegend) of individual cytokines were performed.

### Statistics

In analysis, there was no need for blinding, since all assays were performed using unbiased methodology, including quantitative digital image analysis. All the statistical analysis was performed with GraphPad Prism software version 9 (GraphPad Software, Inc., San Diego, CA). All the data were analyzed using ordinary one-way ANOVA with Dunnet’s correction for multiple comparisons and presented as mean ± SE, unless otherwise specified. A p-value less than 0.05 was deemed statistically significant. Prism software tests for normality, and no outliers were detected throughout our data.

For *in vivo* flow cytometry data for microglia and splenocytes, cell count means of each inflammatory marker were studied. Blood brain barrier permeability data was analyzed by comparing the mean intensity and the mean integrated density of dye extravasation between all groups. The means were determined after the threshold intensity values for detection were established, similar to prior studies in our lab (39–42). For each group, comparisons of the mean concentrations were performed for our individual ELISA and multiple-cytokine assay data. Cytokine ELISA studies were performed with triplicate samples from each cerebral tissue culture.

## Results

### Blood Brain Barrier Permeability

**Figure 2:**
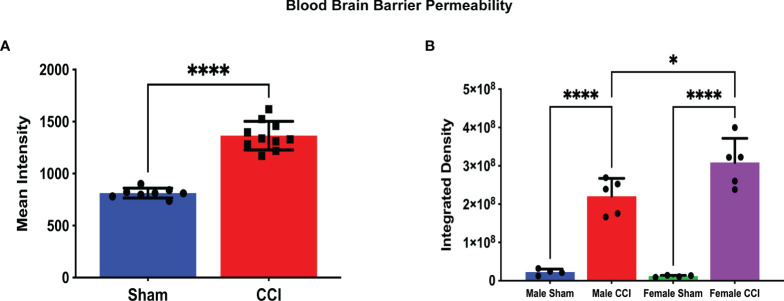
Blood-Brain Barrier Permeability as measured by Alexa Fluor Dye extravasation. In this analysis, a minimum threshold of 1.5k was set for detection of BBB permeability; this threshold was set to eliminate any artifact fluorescence that may be detected by the scanner. The measurements displayed are the mean intensity of dye for all CCI rats compared to all sham rats **(A)** and the mean integrated density for each group **(B)**. There was a significant difference in the mean intensity of dye extravasation when comparing all sham rats to all CCI rats (**A**; CCI mean intensity = 1365 ± 43.61, sham mean intensity = 813.2 ± 16.66, p < 0.0001). The integrated density is the sum of detected fluorescence of dye in a specific area of cerebral tissue. A higher integrated density indicates a more permeable BBB in a specific area of cerebral tissue. Both the female CCI (mean integrated density = 3.08 x 10^8^ ± 2.83 x 10^7^) and the male CCI (2.20 x 10^8^ ± 4.05 x 10^6^) groups recorded higher mean intensities compared to their respective sham group (female sham mean = 1.20 x 10^7^ ± 1.08 x 10^6^, male sham mean = 2.25 ± 4.05 x 10^6^). The female CCI group did record a higher integrated density compared to the male group, and this difference did reach statistical significance (mean difference = 87893520 ± 26653677, p = 0.0210). (****p < 0.0001, *p < 0.05).

In our study of changes in BBB permeability, we did note differences at the 72-hour time point post-injury. When comparing all CCI rats to all sham rats, the mean intensity of detected dye was significantly greater in the CCI group compared to the sham group (**Figure 2A**; CCI mean intensity = 1365 ± 43.6, sham mean intensity = 813.2 ± 16.6). The mean integrated density value of extravasated dye in the CCI groups for both sexes was greater than that of their respective sham groups. This difference was statistically significant (**Figure 2B**; female sham mean = 1.20 x 10^7^ ± 1.08 x 10^6^, female CCI mean = 3.08 x 10^8^ ± 2.83 x 10^7^, p<0.0001; male sham mean = 2.25 x 10^7^ ± 4.05 x 10^6^, male CCI mean = 2.20 x 10^8^ ± 2.08 x 10^7^, p<0.0001).

When comparing the injured groups for each sex, the mean integrated density value of dye extravasation was slightly larger in the female group compared to the male group. This difference was statistically significant (**Figure 2B**; female CCI mean = 3.08 x 10^8^ ± 2.83 x 10^7^, male CCI mean = 2.20 x 10^8^ ± 4.05 x 10^6^, p = 0.0210). When comparing the sham groups based on sex, the mean integrated density value of the male sham group was marginally higher, but this difference did not reach statistical significance (female sham mean = 1.20 x 10^7^ ± 1.08 x 10^6^, male sham mean = 2.25 x 10^7^ ± 4.05 x 10^6^, p=0.7730).

To determine if the size of rats was a confounding factor in this difference, we used a Spearman’s correlation test comparing the integrated density values from each individual rat in relation to their body and brain weight. This analysis was performed to determine if there was a correlation between BBB permeability after injury and weight. In an unpaired t-test, male rats recorded a significantly heavier body weight compared to female rats (Male mean weight = 252.0 ± 2.58, Female rats = 216.9 ± 2.06; **Supplemental Figure 1**). While female CCI rats were significantly smaller in body weight compared to male rats (**Supplemental Figure 2**), there was no statistically significant correlation of body weight with a lower integrated density in all CCI rats (**Supplemental Table 1**). In an unpaired t-test, there was no significant difference in brain weight between male and female rats overall or male and female rats in their respective sham or CCI groups (male brain weight mean = 1.31, female = 1.327, p = 0.64; **Supplemental Figures 3**, **4**). When plotting integrated density values against brain weights of CCI rats, there was no significant correlation observed either (**Supplemental Table 1**).

### Microglia Activation

Brain and spleen samples were obtained 7 days after injury for cell analysis with flow cytometry. Microglia markers included CD45, CD11b/c, P2Y12, CD32, and CD163. On cell counts, CD45, P2Y12, and CD11b cell surface markers were elevated in both the male and female injury groups when compared to their respective sham groups (CD45 male CCI vs male sham mean difference = 237.4 ± 72.8, p = 0.0102; female CCI vs female sham mean difference = 228.6 ± 35.6, p = 0.0135; P2Y12 male CCI vs male sham mean difference = 38.50 ± 2.26, p = 0.0006; female CCI vs female sham mean difference = 38.50 ± 3.07, p < 0.0001; CD11b male CCI vs male sham mean difference = 145.1 ± 33.776, p = 0.0012; female CCI vs female sham mean difference = 150.4 ± 9.91, p = 0.0009). When comparing injury groups based on sex, no significant difference in the mean counts was observed for CD32 (58.92 ± 15.3, p = 0.76), CD45 (0.000 ± 40.1, p > 0.9999), P2Y12 (3.600 ± 2.4, p = 0.9702), CD163 (0.50 ± 2.4), and CD11b (1.400 ± 24.9, p > 0.9999). Statistically significant differences were not observed when comparing the male and female sham groups (**Supplemental Figures 5A, F**).

**Figure 3:**
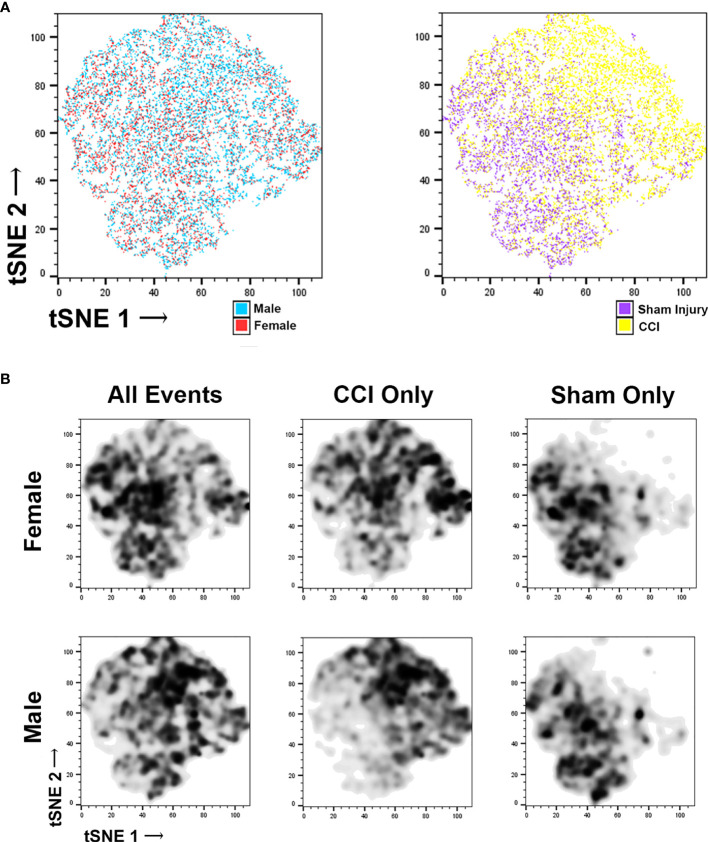
t‐Distributed stochastic neighbor embedding (t-SNE) of microglia flow cytometry data. Each individual data point in the tSNE plot represents individual microglia counted through the flow cytometer. The colors applied to the plots **(A)** allow us to distinguish the microglia based upon their sex (male or female) or their injury (sham or CCI). The colorless plots **(B)** represent density plots of the same microglia data portrayed in the color tSNE plots. The density plots depict specific microglia populations by highlighting the microglia representative of specific experiment groups. When viewing all microglia (CCI and sham injury) separated on the basis of sex, there are specific populations that are clear to distinguish, as expected. When viewing each sex group of only CCI injury, some minor differences are noted, but the microglial populations occupy similar areas, indicating that there are likely more similarities. The density plots of sham groups similarities between the male and female groups as well.

Microglia flow data was further characterized using t-distributed stochastic neighbor embedding (t-SNE). t-SNE uses all data points obtained from flow cytometry for each specific injury group, forming a map of the different cell populations based on cell surface markers (supplemental figure 6A and 6B) and cell morphometry (**Supplemental Figure 6C**). We then coded these data plots to distinguish individual microglia on the basis of sex and injury (**Figure 3A**). This data was then used to make a density plot (**Figure 3B**), which distinguished where the cells from each group localize to on the plot. These plots were then compared it with the cell surface marker and the morphometric analysis plots. In the CCI groups, there is increased density of cell counts in regions corresponding with activated microglia, whereas in the sham groups there is a lower density in these areas. When comparing the female CCI with the male CCI groups, the density plots are similar; some differences exist in that the female CCI group may feature a larger population of typical non-activated microglia compared to the male CCI group.

### Lymphoid and Myeloid Polarization

Spleens were obtained 7 days after injury for cell analysis with flow cytometry. Splenocyte panel included CD3, CD4, CD8, CD45RA, MHC-II, CD8, CD11b, and CD25. This panel allows us to observe changes in the adaptive immune response. When comparing groups, the panel helps us determine differences in T cell subtypes, such as cytotoxic, helper, and regulatory, and peripheral monocytes. The time interval of 7 days post-injury is a commonly used interval for splenocyte activation in our lab, and was selected in order to remain consistent with our prior experiments (41).

Flow cytometry analysis of splenocytes showed no statistically significant differences were observed for CD3, CD4, CD8, and Treg cells when comparing injury animals to sham, male injury to female injury, and male sham to female sham. MHC-II was recorded at higher counts for the male groups compared to the female groups, but these differences did not reach statistical significance **Supplemental Figures 7A–H**).

### Cytokine Analysis

Brain tissue samples were obtained from rats 6 hours post-injury for cytokine analysis, to study for differences in inflammatory cytokine expression in the acute phase after injury. Flow cytometry was utilized for a multiple cytokine assay (BioLegend Legendplex). To complement this, individual cytokine ELISAs were performed for TNF-alpha, IL-1β, and IL-17.

**Figure 4:**
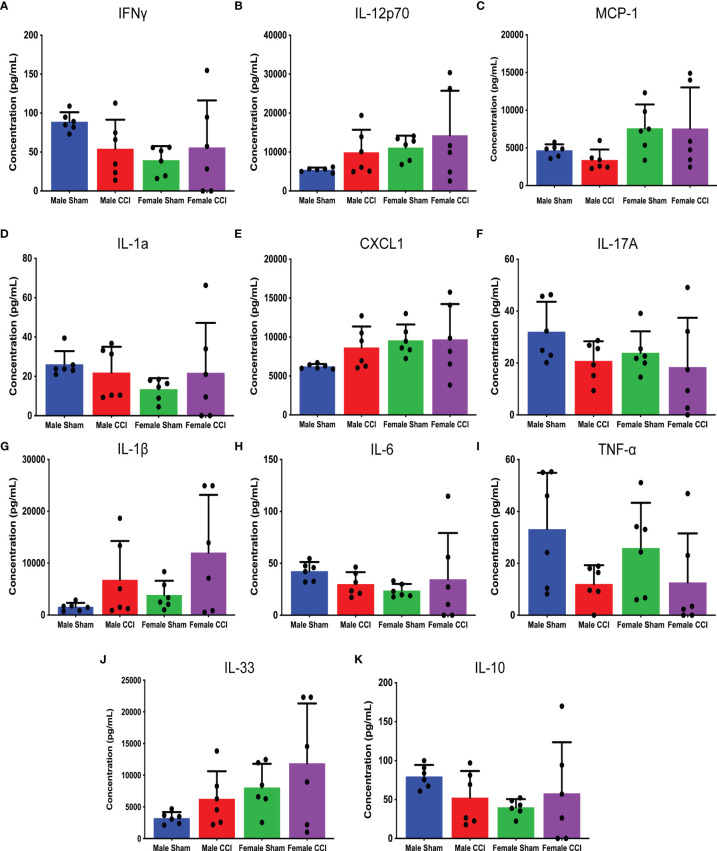
Multiple inflammatory cytokine analysis of cerebral tissue culture. The above charts depict the mean concentrations of individual cytokines in cerebral tissue cultures detected by the cytokine assay. The following cytokines were analyzed in this assay: IFN-γ **(A)**, IL-12p70 **(B)**, MCP-1 **(C)**, IL-1A **(D)**, CXCL-1 **(E)**, IL-17A **(F)**, IL-1β **(G)**, IL-6 **(H)**, TNF-⍺ **(I)**, IL-33 **(J)**, IL-10 **(K)**. While there were multiple differences detected between the male CCI and female CCI groups regarding specific cytokine levels, none of these differences reached statistical significance.

**Figure 5:**
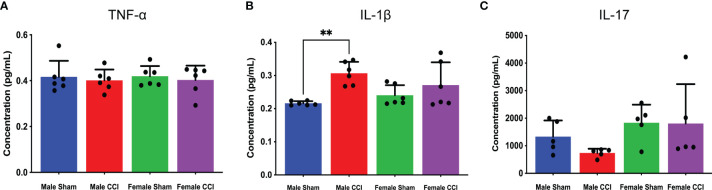
Cytokine ELISAs. The above charts depict the average concentration of TNF-⍺ **(A)**, IL-17 **(B)**, and IL-1β **(C)** detected in cerebral tissue culture from individual ELISAs. TNF-⍺ concentrations were mostly equivalent across all groups. The female groups recorded higher IL-17 concentrations than the male groups, but there was no significant difference between the female CCI group and the female sham group **(B)**. IL-1β concentrations were elevated in the CCI groups, but there was no significant difference between the female CCI or male CCI groups **(C)**. **p < 0.005.

In the analysis of the multiple cytokine assay, none of the mean differences between the male CCI and the female CCI groups reached statistical significance for any cytokine (**Figures 4A–K**). In the individual ELISAs, we found no statistically significant differences between the male and female CCI groups. Male CCI rats recorded a significantly higher concentration of IL-1β than the male sham rats (**Figure 5**; mean difference = 0.09070 ± 0.01, p = 0.0045).

## Discussion

Our data found little difference in neuroinflammatory markers of injury when comparing males and females in a CCI model in the acute time period. These data are important as many have called for evaluating sex as a biological variable, by testing both males and females in experimental protocols. In this study, we sought to compare multiple properties of neuroinflammation between male and female sex, in a TBI rodent model. The properties compared were blood-brain barrier permeability, splenocyte activation, microglial activation, and inflammatory cytokine production. Each study was conducted at certain time intervals after injury (**Figure 1**) to account for the progression of neuroinflammation after a TBI. Time points for each study were selected based on our prior experience with each test.

Splenocyte activation markers did not differ significantly between sexes at 7 days post-injury. Microglial activation markers 7-day post injury had some differences noted on t-SNE, as there may have been more typical non-activated microglia in the female CCI group. However, their density plots did show that this group also had a nearly equivalent number of activated microglia; morphometry of the microglia in male and female CCI groups was largely similar. Our multiple cytokine assay and traditional ELISA showed some minor differences between the male and female groups at the 6-hour time point post injury, but none that reached statistical significance. Of all the studies, only a subset of the BBB permeability analysis showed a significant difference between sexes.

In the analysis of BBB permeability at 72 hours post-injury, the female CCI group recorded a higher mean integrated density compared to the male CCI group, but not a higher mean intensity. This result does suggest that there may be some sex-based difference in how a traumatic injury affects BBB permeability. We considered that the significantly smaller body weights of the female rats may have factored into this, thus the reason for our correlation analysis between weights and integrated density values. However, there was not a statistically significant correlation of body weight with a lower integrated density in all CCI rats. We also looked for correlations between brain weights and BBB permeability after injury, but there were no trends or significant correlations observed. These lack of correlations between brain and body weight and BBB permeability make the potential for weight as a confounding factor unlikely. The result does suggest that BBB permeability may be greater in female rats after CCI, but this was only shown in one specific subset of the BBB permeability analysis, and only after a certain range of fluorescence intensities was set prior to the analysis. One could argue that if this was a true difference on the basis of sex, BBB permeability would be a consistent finding across all analyses using common threshold parameters. In contrast to our results, Julienne et al. used a similar CCI injury model, and did not find a difference in BBB alteration (27). Interestingly, they did find that there may be a difference in vascular repair, as male rats had an increased activation and expression of β-catenin, a marker of angiogenesis and vascular repair, as well as an increase in cortical vascular density (27). A recent study by Bharadwaj et al. found results regarding BBB permeability similar to ours. In a CCI-model of mice, they found that females had a greater extravasation of a macromolecular HRP tracer at 3 hours and 24 hours post injury, compared to males; they did not observe a difference in neuroglial response (28). An earlier study by Duvdevani et al. used a CCI rodent model as well, using Evans Blue dye to evaluate BBB permeability; they found no significant sex difference in BBB permeability, and no effect of hormonal status at 1 day post injury (29). O’Connor et al. found that BBB permeability may be elevated in male rats after a diffuse injury. In a frontal impact diffuse TBI injury model, O’Connor et al. found an increase in Evans Blue dye extravasation in male rats compared to female rats. Interestingly, administration of exogenous estrogen or progesterone reduced BBB permeability in males, and also attenuated the increase in cerebral edema post-TBI in males and ovariectomized females (21).

Our study does have limitations. We utilized a sham-injury model that does not include a craniectomy, as studies have found that craniectomy procedures create a risk of confounding injury to the brain and cerebral blood vessels and our study largely focuses on the difference between male and female CCI-injured groups (37, 38). In each individual experiment, we studied changes in neuroinflammation and TBI pathology at specified time points. These time points were selected based on prior studies using these tests in our lab and upon our interpretation of discussions in the literature of the importance of certain time points in the progression of neuroinflammation in TBI. It is possible however, that differences may be seen at different time points in each individual test. While the male and female rats were of equal age, we did not coordinate the injury to occur at a specific stage of estrous cycle of female rats. There was some variability in the female rat cytokine analysis, and it is possible that this variability may be due to individual rats being at various stages of the estrous cycle. However, it is worth noting that this variability was not limited to the female rats, as it was also noted in certain other neuroinflammatory markers for the male rat groups. Some markers of flow cytometry, such as CD45 and CD11b appear to have larger variability in the male CCI groups compared to the female groups. For this study, we selected time points used in previous studies of neuroinflammation following CCI in rats, however, there is a reasonable possibility that our assays and time points were not sufficient to capture dynamic or temporal differences between male and females in TBI.

Our study sought to elucidate whether there were clear and significant differences in outcomes of neuroinflammation in our CCI-model between male and female rats. As stated above, the only significant difference detected was a potential increase in BBB permeability. In other pre-clinical studies, it seems that potential sex-based differences are varied. These differences can extend to multiple different features of neuroinflammation, and may vary with different models of TBI (**Table 2**). Similar to the human studies, pre-clinical animal studies have yielded varying results. Neurodegeneration in rats after TBI may evolve at different rates in each sex, but it does not appear that one sex incurs a higher level of neurodegeneration (30, 31). In a series of experiments with piglets, Armstead et al. found that cerebral autoregulation males may be impaired after FPI, which is thought to be due to an upregulation of endothelin-1 (15–17). In contrast to our findings regarding microglia, Doran et al. found some functional differences between sexes in rats sustaining CCI injury. After CCI injury, microglia from female rats had decreased phagocytic activity and production of reactive oxygen species; however, they found no sex-based difference in the expression of pro-inflammatory cytokine expression by microglia (14). Villapol et al. found results that were similar; in their rodent CCI-model, there was a faster influx and activation of microglia in males compared to females, but the differences became indistinguishable after 1-week post-injury (32). Some studies have also indicated that it may be females with a more rapid pro-inflammatory response. In a meta-analysis of their data from a closed head injury of mice, Späni et al. found that females had a greater increase in pro-inflammatory markers like IL-1β, IL-6, and TNF-α in the 6-hour window post-injury; they also found that the anti-inflammatory cytokine IL-10 was elevated in males, but not females (51). In a CCI rat model, Taylor et al. found that ovariectomized females had an increased expression of IL-1β and IL-6 in the ipsilateral hippocampus, but males had a higher expression of TNF-α (22).

As mentioned previously, one limitation of our study is that we did not control for the stage of estrous cycle in female rats. However, the literature on the relationship between hormones and pathology presents various findings. Certain levels of circulating endogenous hormones have been linked to improved outcomes in TBI. Roof et al. found that pro-estrous female rats recorded a lower brain edema content at injury site compared to males. This difference was even greater in female rats with induced pseudopregnancy. They found that the decrease in cerebral edema after injury was more likely due to progesterone and not estrogen, as this decrease was not seen in ovariectomized rats until progesterone supplementation was administered (23). In a diffuse TBI model, Roof et al. found that cortical blood flow (CBF) decreased in male rats at a greater rate than females after injury; CBF was lower in ovariectomized females compared to intact females. However, the decrease of CBF in ovariectomized rats was not corrected with the administration of estrogen replacement (24). As mentioned above, Duvdevani et al. did not find an effect by hormonal status on BBB permeability (29), whereas O’Connor et al. found a treatment effect of estrogen and progesterone on reducing BBB permeability in male rats (21).

In another TBI model using ovariectomized rats, Bramlett et al. showed that intact female rats had smaller contusion volumes compared to male rats and ovariectomized female rats. Neuroprotection in this specific study was not limited to a specific stage of the estrous cycle, as the average contusion volume was similar between pro-estrous and non-pro-estrous female rats (25). Wagner et al. found that female rats achieved better functional outcomes, but the difference did not depend on estrous cycle stage. Using the CCI-injury model, Wagner et al. found that regardless of estrous phase, female rats performed better than male rats in motor performance tests. However, that difference in behavioral performance was not influenced by serum estrogen and progesterone levels at the time of injury (26).

Interestingly, there is some literature that supports the notion that it is actually the injury itself that induces disruption of hormonal signaling. In an observational human study, Wagner et al. found that progesterone levels increased in male patients after injury, and in female patients the progesterone level was equivalent to control patients in the luteal or follicular phase of the menstrual cycle. They found that differences in hormone levels between the male and female groups were small and did not reach significance. In their combined study sample of male and female patients, high progesterone levels post-injury were associated with an increase in acute care mortality (18).. This does raise the question of whether the endogenous hormones alone or the disruptions in the hormone signaling due to cerebral injury create these potential differences in potential outcomes. Follow-up studies with TBI patients have found that injury is associated with chronic hypopituitarism (19, 20). Amongst all of this, testosterone may also need to be considered, as it can be an estradiol metabolite. In the previously mentioned study by Wagner et al., they reported that testosterone levels in male patients were lower after TBI compared to their controls, but in females, testosterone was elevated after injury. As with progesterone, they found a similar association with increased mortality in the patients that recorded elevated testosterone levels after injury (18). What our study does not evaluate is the potential response to any given treatment or intervention which may be influenced by sex of the animal.

Retrospective studies of sex-based differences in TBI have produced various findings of mortality rates after TBI (**Table 1**). A large retrospective clinical study of patients in the Chinese Head Trauma Data Bank of TBI was conducted by the Chinese Head Trauma Study Collaborators. In their study of 7145 acute TBI patients, the mortality rate in males and females was similar. When the study group stratified for severe TBI, they again found similar mortality rates (12). In another large retrospective study of 72,294 patients with moderate to severe TBI, males were compared to females. There was a lower risk in mortality in female patients overall, but when the female patients were stratified, it was found that this difference was present in perimenopausal or post-menopausal females. There was no difference in mortality when comparing pre-menopausal female patients to males, downplaying the influence of endogenous hormones on TBI outcomes (5).

Our results fit with the current literature discussing the potential influence of sex on TBI (**Tables 1**, **2**). Some sex-based differences have been noted in numerous studies utilizing various injury models. However, a distinct and common difference that is consistent across pre-clinical models has not been established in the most current literature. As our lab and others have discussed in prior reviews, the sex differences that may occur in TBI in the pre-clinical and clinical settings are wide, varied, and appear to have some dependence on the model or mechanism of TBI (51–53). TBI outcomes based on sex can also be dependent on the specific outcomes studied. In relation to our own model of TBI, our results indicate that the difference in outcomes between male and female rats may be minor, and difficult to control. In relating this to our review of the literature, further controlling our model on the basis of the hormonal balance or sex of rats may only yield small differences. These small differences may not reflect a significant or true variation in the progression of neuroinflammation based upon our preferred markers.

## Data Availability Statement

The original contributions presented in the study are included in the article/**Supplementary Material**. Further inquiries can be directed to the corresponding authors.

## Ethics Statement

The animal study was reviewed and approved by the Animal Welfare Committee University of Texas Health Science Center at Houston, TX, USA.

## Author Contributions

MS and KP wrote the main manuscript text and prepared figures. MS and SO prepared **Figures 1**–**5**. AW prepared **Tables 1** and **2**. The manuscripts was reviewed by all authors. All authors contributed to the article and approved the submitted version.

## Funding

MS was supported by the National Institute of General Medical Sciences of the National Institutes of Health under award number 2T32GM008792. SO was supported by the National Institute of Neurological Disorders and Stroke under award number R21NS116302. CC and SO have received research support from Athersys, CBR Systems, Hope Bio, and Biostage. The funders were not involved in the study design, collection, analysis, interpretation of data, the writing of this article or the decision to submit it for publication.

## Conflict of Interest

CC is on the Scientific Advisory Board of Cellvation and CBR.

The remaining authors declare that the research was conducted in the absence of any commercial or financial relationships that could be construed as a potential conflict of interest.

## Publisher’s Note

All claims expressed in this article are solely those of the authors and do not necessarily represent those of their affiliated organizations, or those of the publisher, the editors and the reviewers. Any product that may be evaluated in this article, or claim that may be made by its manufacturer, is not guaranteed or endorsed by the publisher.

## Supplementary Material

Supplementary Figure 1 Mean body weights of male and female rat groups in BBB permeability study combined with statistical analysis.

Supplementary Figure 2 Subgroup analysis of male and female body weights in BBB permeability study with statistical analysis.

Supplementary Figure 3 Subgroup analysis of male and female brain weights in BBB permeability study with statistical analysis.

Supplementary Figure 4 Mean brain weights of male and female rat groups combined in BBB permeability study with statistical analysis.

Supplementary Figure 5 Microglial traditional flow cytometry. The above charts depict the mean value of counted microglial cells expressing CD45 **(A)**, CD11b **(B)**, P2Y12 **(C)**, CD32 **(D)**, CD163 **(E)**, and RT1b **(F)**. No significant changes were noted between sham and injury in the number of cells expressing CD32. Male and female CCI groups recorded higher mean counts of cells expressing CD45, CD11b, and P2Y12 compared to their respective sham groups. No significant differences were detected when comparing the female CCI group to the male CCI group for any of the microglial activation markers.

Supplementary Figure 6 t‐Distributed stochastic neighbor embedding (t-SNE) of microglia flow cytometry data. These specific plots are coded to identify cells from the ipsilateral cerebral hemisphere, in particular microglia, on the basis of cell surface markers **(A)**, CD45 and P2Y12 expression **(B)**, and size by morphometric analysis **(C)**.

Supplementary Figure 7 Splenocyte traditional flow cytometry. The above charts depict the mean counts of cells derived from splenic tissue with the following marker expression: CD3-CD45RA+ **(A)**, CD3+CD45RA- **(E)**, CD11b **(C)**, CD4 **(D)**, MHC-II **(F)**, CD3 **(G)**, CD8 **(H)**. Regulatory type T cells are also identified *via* flow cytometry **(B)**. While the male CCI group did record a higher mean count for each of these markers, none of the differences reached statistical significance.

Supplementary Table 2 Raw data of rat body/brain weights and BBB permeability mean fluorescent intensity and integrated density.
