# Supplementary figures and images for: Determining Sex-Based Differences in Inflammatory Response in an Experimental Traumatic Brain Injury Model
Source: Front Immunol. 2022 Feb 9;13:753570. doi: 10.3389/fimmu.2022.753570 (PMC8864286; doi:10.3389/fimmu.2022.753570)

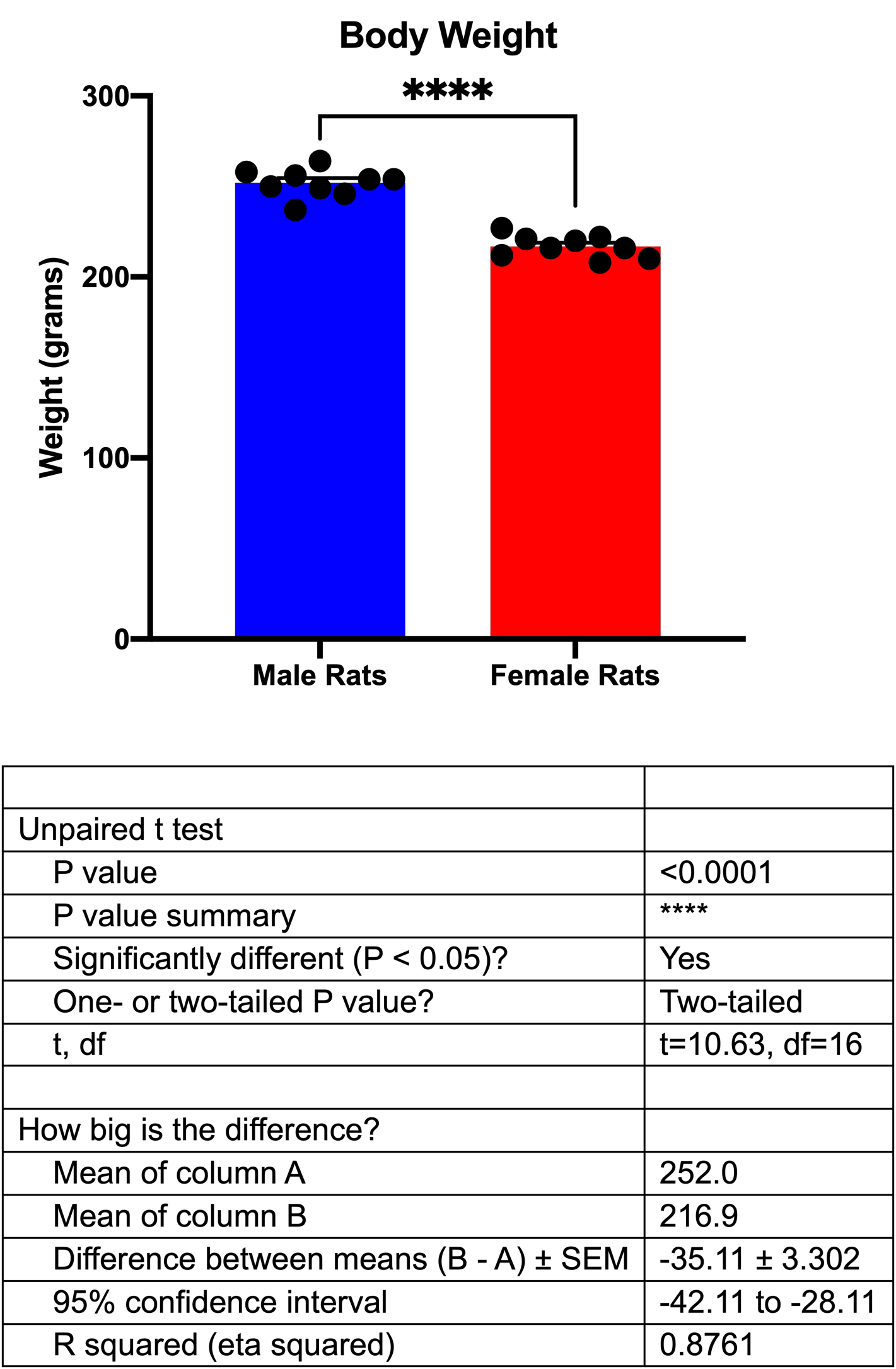

Supplement: Supplementary Figure 1 — Mean body weights of male and female rat groups in BBB permeability study combined with statistical analysis. [file Image_1.tiff]

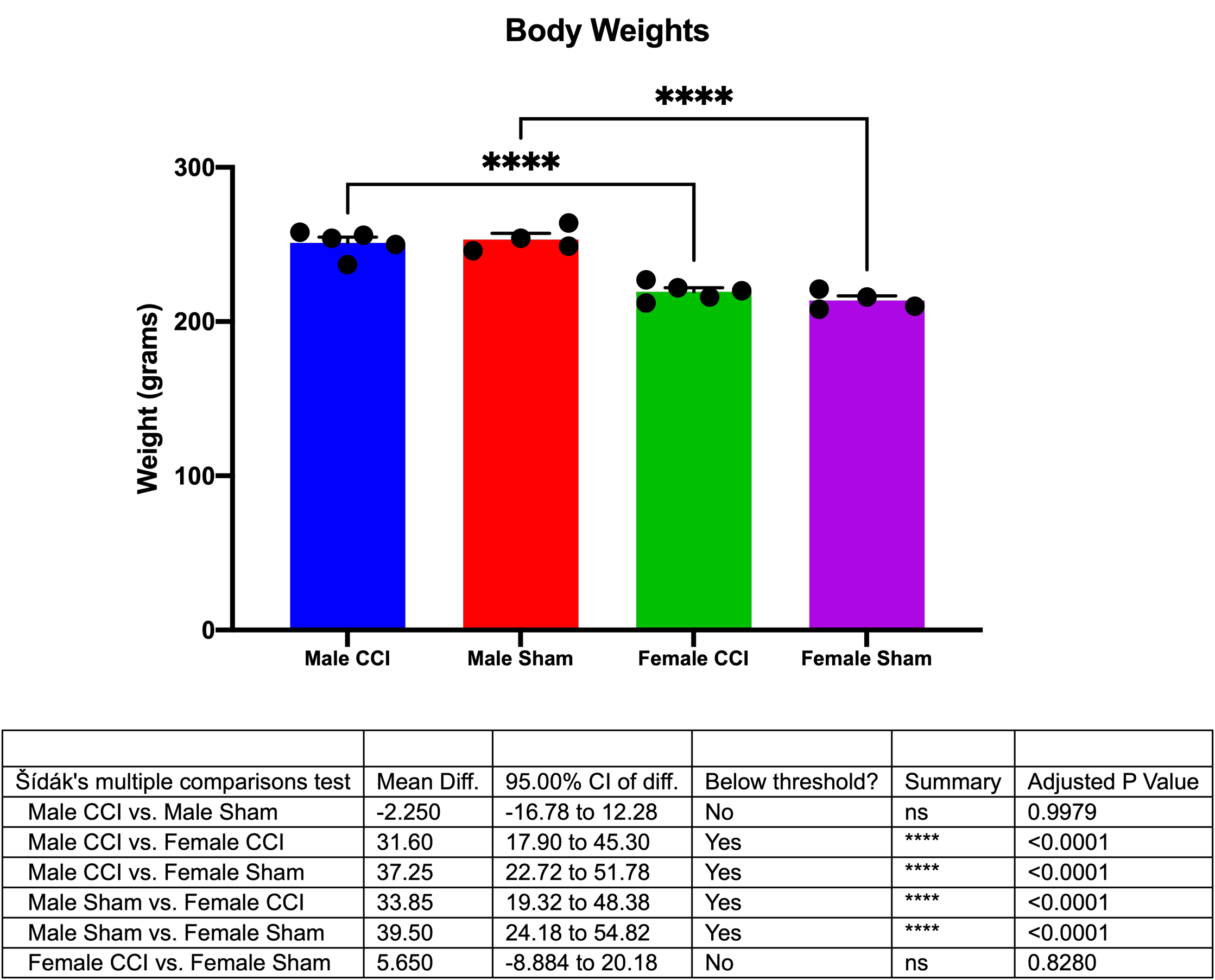

Supplement: Supplementary Figure 2 — Subgroup analysis of male and female body weights in BBB permeability study with statistical analysis. [file Image_2.tiff]

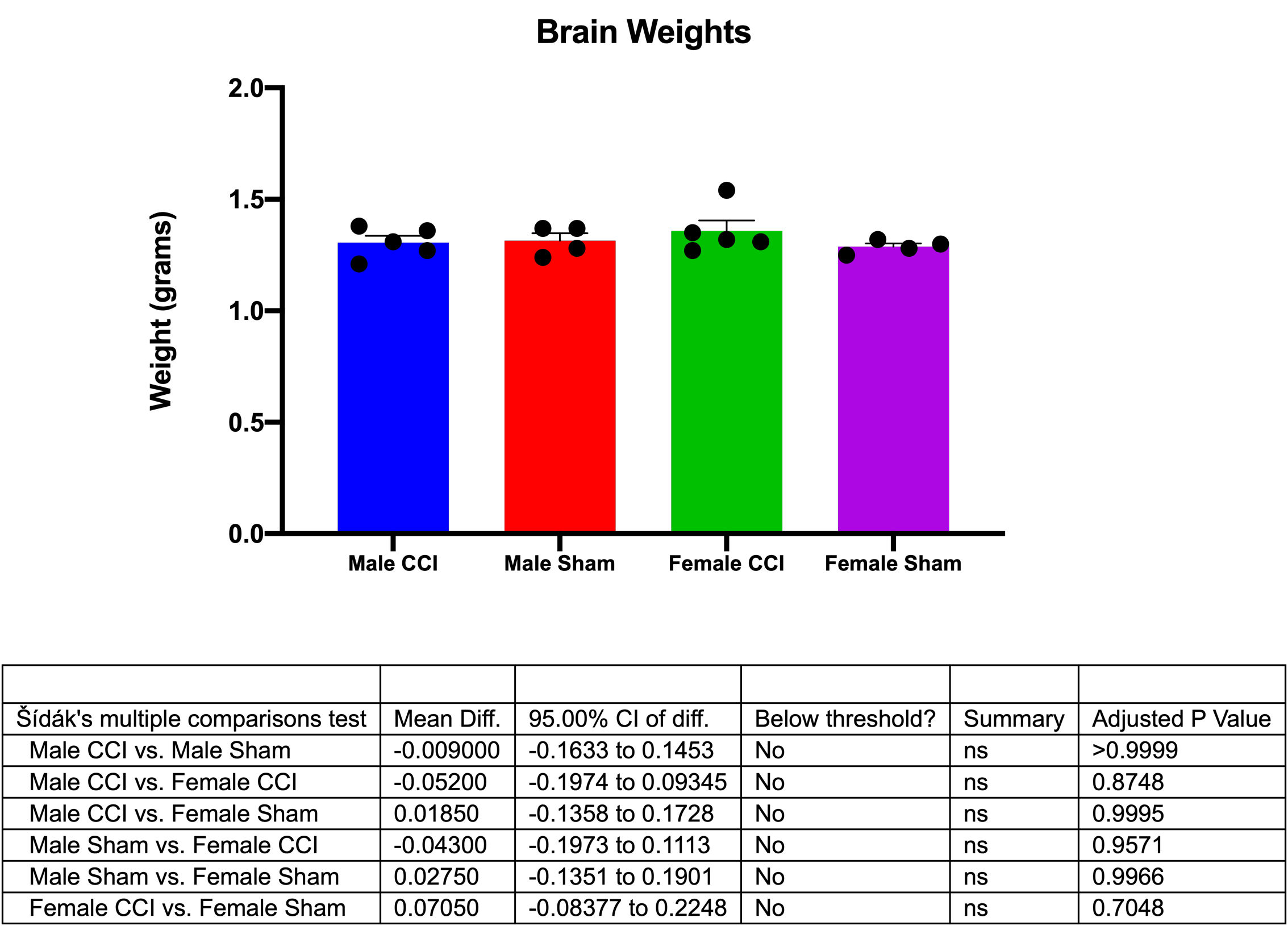

Supplement: Supplementary Figure 3 — Subgroup analysis of male and female brain weights in BBB permeability study with statistical analysis. [file Image_3.tiff]

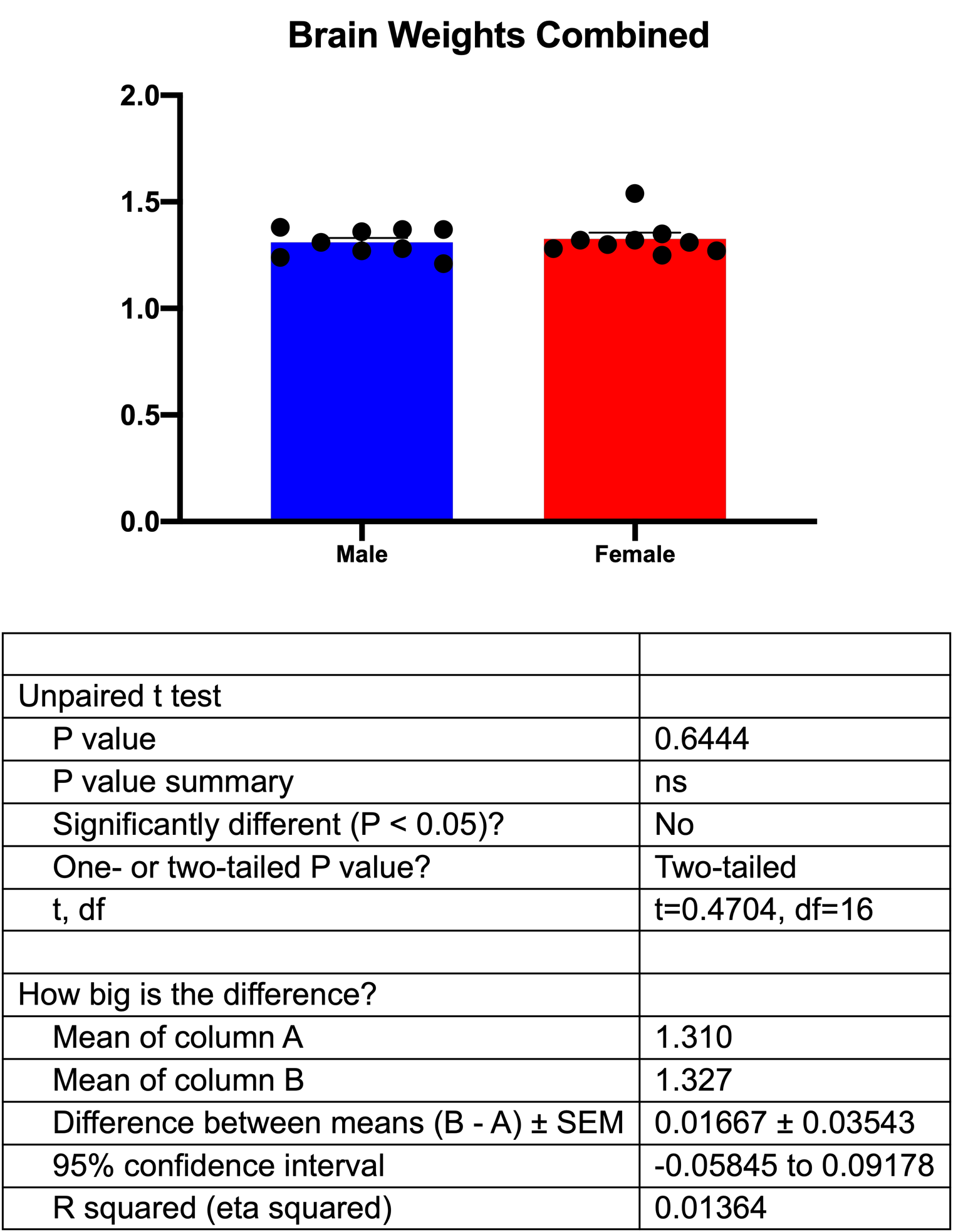

Supplement: Supplementary Figure 4 — Mean brain weights of male and female rat groups combined in BBB permeability study with statistical analysis. [file Image_4.tiff]

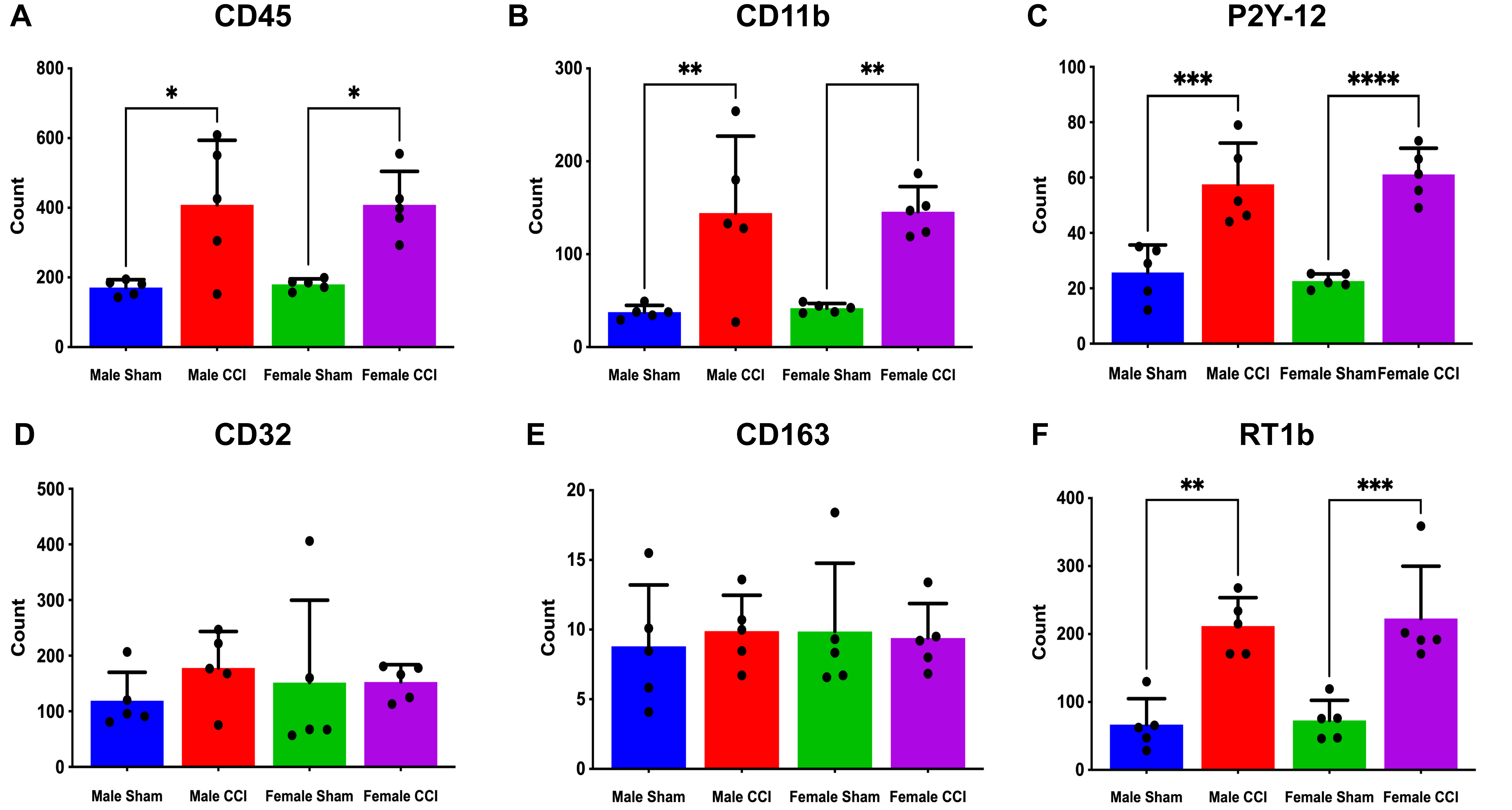

Supplement: Supplementary Figure 5 — Microglial traditional flow cytometry. The above charts depict the mean value of counted microglial cells expressing CD45 (A), CD11b (B), P2Y12 (C), CD32 (D), CD163 (E), and RT1b (F). No significant changes were noted between sham and injury in the number of cells expressing CD32. Male and female CCI groups recorded higher mean counts of cells expressing CD45, CD11b, and P2Y12 compared to their respective sham groups. No significant differences were detected when comparing the female CCI group to the male CCI group for any of the microglial activation markers. [file Image_5.tif]

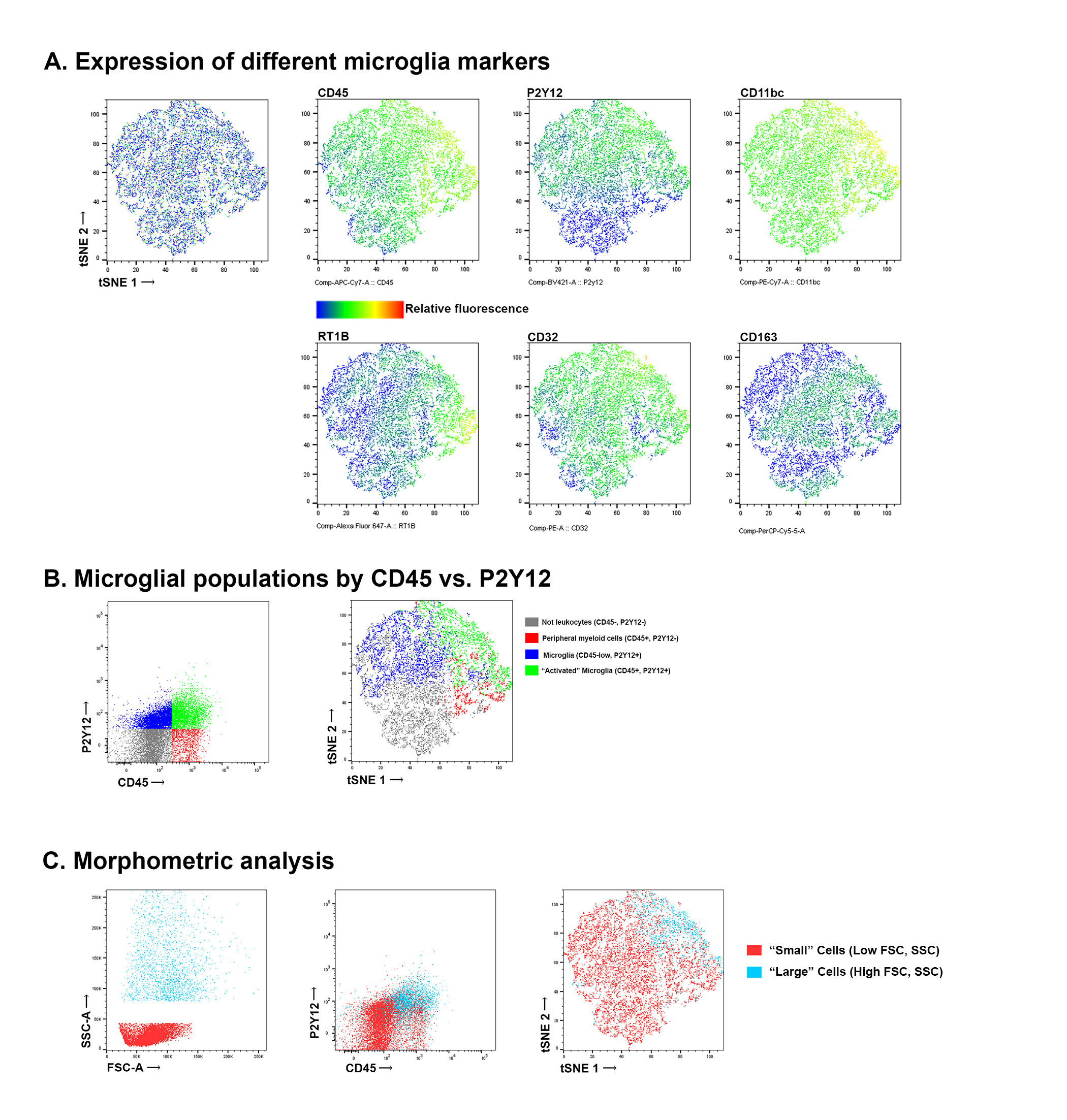

Supplement: Supplementary Figure 6 — t‐Distributed stochastic neighbor embedding (t-SNE) of microglia flow cytometry data. These specific plots are coded to identify cells from the ipsilateral cerebral hemisphere, in particular microglia, on the basis of cell surface markers (A), CD45 and P2Y12 expression (B), and size by morphometric analysis (C). [file Image_6.tif]

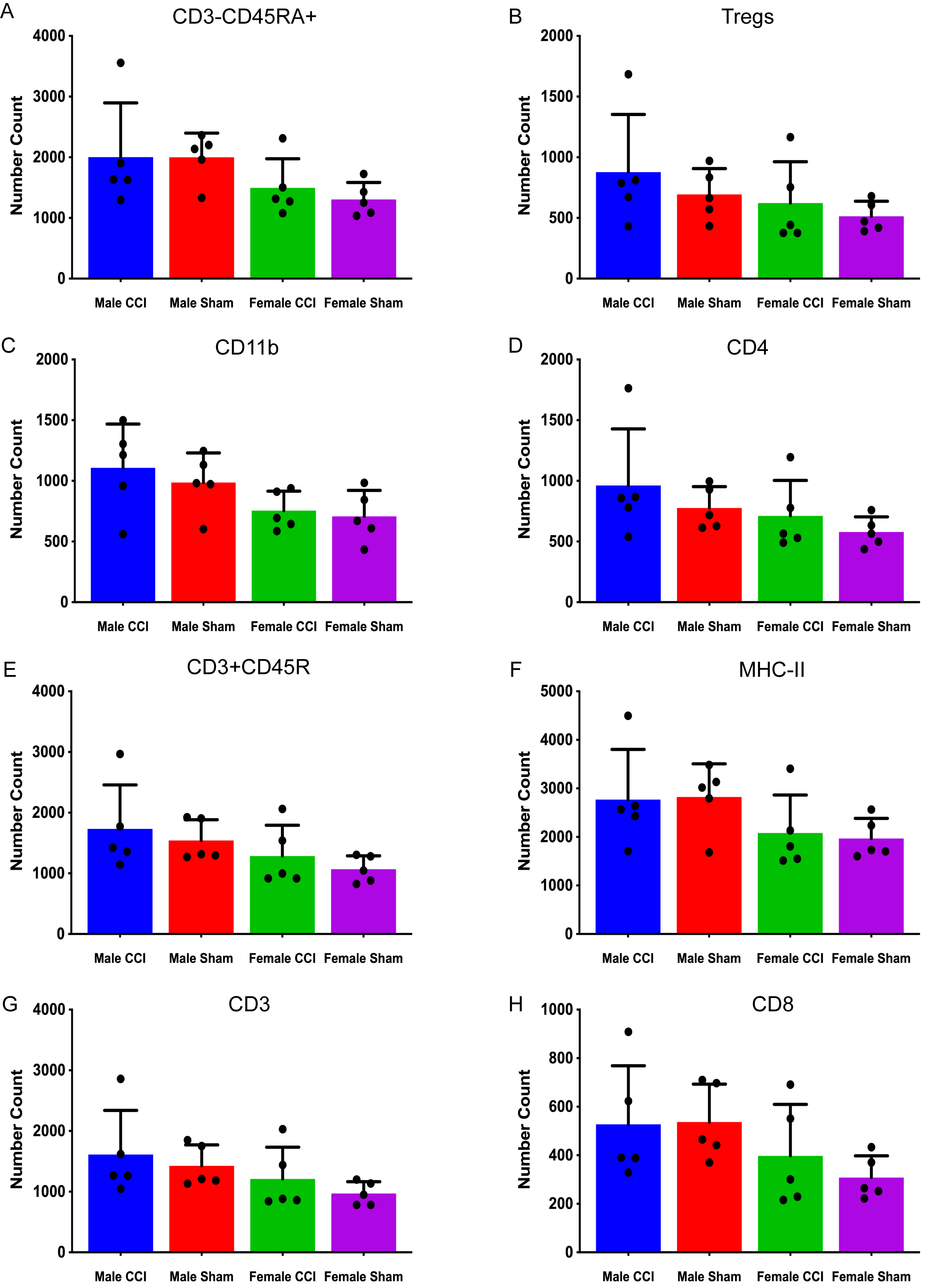

Supplement: Supplementary Figure 7 — Splenocyte traditional flow cytometry. The above charts depict the mean counts of cells derived from splenic tissue with the following marker expression: CD3-CD45RA+ (A), CD3+CD45RA- (E), CD11b (C), CD4 (D), MHC-II (F), CD3 (G), CD8 (H). Regulatory type T cells are also identified via flow cytometry (B). While the male CCI group did record a higher mean count for each of these markers, none of the differences reached statistical significance. [file Image_7.tif]
