# Supplementary material for: Determining Sex-Based Differences in Inflammatory Response in an Experimental Traumatic Brain Injury Model
Source: Front Immunol. 2022 Feb 9;13:753570. doi: 10.3389/fimmu.2022.753570 (PMC8864286; doi:10.3389/fimmu.2022.753570)
Supplement: Supplementary file 8 [file Table_1.docx]

|  |  | Mean ± SD | | | |  |
| --- | --- | --- | --- | --- | --- | --- |
|  | Cytokine / Cell Surface Marker | Female Sham | Female CCI | Male Sham | Male CCI | N for 95% Power |
| Cytokine | MCP-1 | 7601.15 ± 3168.68 | 7570.02 ± 5464.60 | 4677.00 ± 799.65 | 3395.07 ± 1387.76 | 10 |
|  | IL-1β | 3863 ± 2744 | 12059 ± 11122 | 1582 ± 778.9 | 6795 ± 7495 | 86 |
|  | IL-10 | 39.98 ± 10.66 | 57.93 ± 65.69 | 79.73 ± 14.70 | 52.42 ± 34.27 | 2351 |
|  | IFN-γ | 39.54 ± 18.09 | 55.84 ± 60.52 | 88.80 ± 12.30 | 54.23 ± 37.29 | 25334 |
|  | CXCL-1 | 9567 ± 2047 | 9708 ± 4534 | 6225 ± 315.3 | 8672 ± 2693 | 338 |
|  | TNF-α | 25.91 ± 17.43 | 12.62 ± 18.93 | 33.20 ± 21.69 | 12.04 ± 7.24 | 15868 |
|  | IL-12p70 | 11138 ± 3072 | 14283 ± 11465 | 5419 ± 565.5 | 9922 ± 5806 | 114 |
|  | IL-17a | 23.95 ± 8.28 | 18.46 ± 18.98 | 32.08 ± 11.53 | 20.82 ± 7.57 | 976 |
|  | IL-33 | 8052 ± 3759 | 11886 ± 9440 | 3235 ± 932.9 | 6284 ± 4341 | 46 |
|  | IL-1a | 13.48 ± 5.59 | 21.82 ± 25.42 | 26.15 ± 6.71 | 21.93 ± 13.11 | 878539 |
|  | IL-6 | 23.75 ± 6.40 | 34.74 ± 44.52 | 42.48 ± 8.83 | 30.08 ± 11.52 | 1267 |
| Microglia | CD11b | 41.98 ± 4.92 | 145.8 ± 27.07 | 37.66 ± 7.32 | 144.4 ± 82.86 | 50376 |
|  | RT1b | 72.56 ± 29.76 | 189.0 ± 12.99 | 66.74 ± 38.30 | 211.8 ± 41.81 | 794 |
|  | P2Y12 | 22.66 ± 2.58 | 61.16 ± 9.44 | 25.70 ± 9.88 | 57.56 ± 14.93 | 314 |
|  | CD32 | 151.6 ± 148.3 | 152.6 ± 31.47 | 119.0 ± 51.24 | 177.9 ± 65.77 | 109 |
| Splenocytes | CD11b | 707.0 ± 213.0 | 755.2 ± 159.9 | 987.0 ± 242.8 | 1107 ± 362.0 | 18 |
|  | CD3 | 970.0 ± 194.5 | 1211 ± 522.3 | 1425 ± 345.2 | 1611 ± 727.6 | 67 |
|  | CD4 | 578.6 ± 124.6 | 711.8 ± 292.1 | 776.8 ± 175.5 | 962.0 ± 466.9 | 64 |
|  | CD8 | 308.4 ± 89.53 | 397.6 ± 212.3 | 536.8 ± 156.2 | 527.8 ± 241.1 | 81 |
|  | Tregs | 513.6 ± 124.2 | 623.2 ± 341.0 | 694.8 ± 212.1 | 877.4 ± 475.4 | 70 |
|  | MHC-II | 1968 ± 413.9 | 2079 ± 781.5 | 2823 ± 684.0 | 2768 ± 1035 | 48 |
|  | CD3-CD45RA+ | 1305 ± 280.0 | 1497 ± 480.4 | 2000 ± 399.7 | 2003 ± 894.7 | 54 |
|  | CD3+CD45RA- | 1068 ± 220.8 | 1286 ± 505.3 | 1542 ± 341.5 | 1733 ± 725.6 | 52 |

**Supplemental Table 1.** A priori power analysis (G*Power 3.1) of cytokines and cell surface markers of microglia and splenocytes. In this analysis, we attempt to show the approximate N values required to achieve 95% statistical power for each individual test.
