# Supplementary material for: Determining Sex-Based Differences in Inflammatory Response in an Experimental Traumatic Brain Injury Model
Source: Front Immunol. 2022 Feb 9;13:753570. doi: 10.3389/fimmu.2022.753570 (PMC8864286; doi:10.3389/fimmu.2022.753570)
Supplement: Supplementary Table 2 — Raw data of rat body/brain weights and BBB permeability mean fluorescent intensity and integrated density. [file Table_2.docx]

| Rat | Desc | Sex | Injury | Body Weight | Brain Weight | Mean FL | Area | Int. Density |
| --- | --- | --- | --- | --- | --- | --- | --- | --- |
| 1 | F Sham | F | Sham | 208 | 1.25 | 737.915 | 59001 | 9458177 |
| 2 | F Sham | F | Sham | 210 | 1.3 | 811.836 | 86538 | 14117696 |
| 3 | F Sham | F | Sham | 216 | 1.32 | 780.63 | 62900 | 10897103 |
| 4 | F Sham | F | Sham | 221 | 1.28 | 823.295 | 78188 | 13435180 |
| 5 | F CCI | F | CCI | 216 | 1.31 | 1524.504 | 211806 | 3.22E+08 |
| 6 | F CCI | F | CCI | 212 | 1.27 | 1310.391 | 185748 | 2.38E+08 |
| 7 | F CCI | F | CCI | 222 | 1.35 | 1338.15 | 188180 | 2.6E+08 |
| 8 | F CCI | F | CCI | 220 | 1.54 | 1459.27 | 208521 | 3.22E+08 |
| 9 | F CCI | F | CCI | 227 | 1.32 | 1619.416 | 223307 | 4E+08 |
| 10 | M Sham | M | Sham | 246 | 1.24 | 901.128 | 108179 | 32054771 |
| 11 | M Sham | M | Sham | 264 | 1.28 | 811.512 | 71134 | 12552122 |
| 12 | M Sham | M | Sham | 249 | 1.37 | 798.249 | 56678 | 20905844 |
| 13 | M Sham | M | Sham | 254 | 1.37 | 840.772 | 85467 | 24418883 |
| 14 | M CCI | M | CCI | 258 | 1.36 | 1219.649 | 186469 | 1.76E+08 |
| 15 | M CCI | M | CCI | 254 | 1.31 | 1280.57 | 167552 | 2.39E+08 |
| 16 | M CCI | M | CCI | 237 | 1.21 | 1398.203 | 248911 | 2.69E+08 |
| 17 | M CCI | M | CCI | 256 | 1.27 | 1329.861 | 169097 | 2.53E+08 |

**Supplemental Table 2.** Raw data of rat body/brain weights and BBB permeability mean fluorescent intensity and integrated density.
